# Prevalence of HIV, HBV, HCV, HPV and syphilis among female sex workers in Kurdistan, west of Iran

**DOI:** 10.22088/cjim.15.1.3

**Published:** 2024

**Authors:** Somaye Mafakheri Bashmaq, Amjad Ahmadi, Behzad Mohsenpour, Khaled Rahmani, Modabber Arasteh, Narges Shams Alizadeh, Asrin Babahajian, Shoaib Advay, Asefeh Abbaszadeh

**Affiliations:** 1 Student Reaserch Committee, Kurdistan University of Medical Sciences, Sanandaj, Iran.; 2 Department of Microbiology, School of Medicine, Hamadan University of Medical Sciences, Hamadan, Iran.; 3 Department of Infectious Disease, Faculty of Medicine, Kurdistan University of Medical Sciences, Sanandaj, Iran; 4 Liver and Digestive Research Center, Research Institute for Health Development, Kurdistan University of Medical Sciences, Sanandaj, Iran.; 5 Department of Psychology, Faculty of Medicine, Kurdistan University of Medical Sciences, Sanandaj, Iran.; 6 Department of Virology, Faculty of Medicine, Kerman University of Medical Sciences, Kerman, Iran.; ¥ Mafakheri Bashmaq and Ahmadi contributed equally in this article

**Keywords:** Prevalence, Female sex workers, HIV, Sexually transmitted infection, Iran.

## Abstract

**Background:**

Female sex workers (FSWs) in most societies run a high risk of health problems, including sexually transmitted infections (STIs) such as viral infections and syphilis. The present study examines the prevalence of viral infections and syphilis among FSWs.

**Methods:**

This cross-sectional study recruited 100 female sex workers (April 2019 to April 2020) who visited the Counseling Center for Behavioral Diseases or were selected via purposeful (snowball) sampling. A questionnaire (demographic information and STI risk factors) was completed in a face-to-face interview with the participants. Blood samples were then taken to test the markers for HBV, HCV Ab, HIV Ab, Rapid Plasma Reagin (RPR) for syphilis and a PCR was taken to test for HPV (in vaginal sex workers from the cervix and anal sex workers from the anal region). The data were analyzed in Stata 14.

**Results:**

Among 100 FSWs, 6 (6%) were infected with HIV, 1 (1%) with hepatitis B, and 2 (2%) were anti-HCV positive. 1 (1%) participant was suspected of having syphilis. Based on the PCR tests, 16 (16%) participants were infected with HPV. Moreover, 68 (68%) FSWs reported having unprotected sex.

**Conclusion:**

Due to the prevalence of viral infections and syphilis and unprotected sex in FSWs, immediate preventive measures are critical for this vulnerable group to control the transmission of these viral infections in society.

Female sex workers (FSWs) in different societies are exposed to social harms, including poverty, violence, exploitation, discrimination, drug use, and health-related problems, including unintended pregnancy and sexually transmitted infections (STIs) such as viral and bacterial infections (1). More than 30 different bacteria, viruses and parasites are known to be transmitted through sexual contact. Eight of these pathogens are linked to the greatest incidence of sexually transmitted disease. Of these, 4 are currently curable: syphilis, gonorrhea, chlamydia, and trichomoniasis. The other 4 are viral infections that are incurable: Hepatitis B, herpes simplex virus (HSV), human immunodeficiency virus (HIV), and human papillomavirus (HPV). STIs are spread predominantly by sexual contact, including vaginal, anal, and oral sex. Some STIs can also be transmitted from mother-to-child during pregnancy, childbirth, and breastfeeding (2, 3). The prevalence of syphilis has risen sharply in the United States over the past decade. More than 30,000 cases of syphilis were reported in 2017 at a rate of 9.5 cases per 100,000 populations. Syphilis is 8 times more common in men and is more common in the age range of 20 to 29 years. The disease is caused by the spirochete *Treponema pallidum* and is transmitted through sexual contact and exposure to genital sores (4, 5). 

About 15% of HIV cases are globally attributed to FSWs who have unprotected sex (6). Poor socioeconomic status, criminalization of their job, multiple sexual partners, having no choice about using condoms, and poor access to proper lubricants increase occupational hazards and the prevalence of STIs in FSWs (7). The prevalence of prostitution in girls above the age of 15 years is estimated at 0.4–4.3 in Africa, 0.2–2.6 in Asia, and 0.1–1.4 in Europe (8). About one-eighth (11.8%) of the FSWs in developing countries are estimated to be infected with HIV, while only 58% of these women have access to healthcare services for HIV and other STIs (9, 10).

Prostitution is illegal and a crime in Iran, and there is no accurate statistics on its prevalence but in a study in 2017 indicated that the population size of FSW in all provincial capital cities in Iran was estimated to be 130,800 (95% UIs: 87,800–168,200). Applying the same proportion to all other major cities, our best estimate for the population size of FSW in urban Iran is 228,700 (95% UIs: 153,500–294,300) (11-13). Due to the increasing transmission of STIs through unprotected sex in Iran, the evaluation of FSWs as a high-risk group with frequent unprotected sex is necessary to control the epidemic of HIV and other STIs. The present study, therefore, examined the prevalence of viral infections and Syphilis among FSWs.

## Methods

**Study design, setting and population:** This descriptive cross-sectional study was conducted over one year from April 2019 to April 2020. 100 FSW women in Sanandaj City (between April 2019 and April 2020) who visited the Counseling Center for Behavioral Diseases were entered into the study via purposeful (snowball) sampling method. The study population comprised women who had sex with at least one client in the past year in exchange for money, drugs, or any goods or services (food, a place to live, etc.). Women who did not consent to participation, blood test, or pap smear test were excluded. 

**Ethical aspects:** This study was approved by the Ethics Committee of the Kurdistan University of Medical Sciences (IR.MUK.REC.1397.017) and in collaboration with the Welfare Organization and the Deputy for Health of the university. At the outset, the participants were briefed about the objective of the study, the type and method of performing the tests. Participants who were willing to participate would then fill out and sign the informed consent form. The participants were not deprived of any facilities if they did not consent to participate. As the patients had a low economic and cultural status, an infectious disease specialist was arranged to provide free-of-charge visits and some therapeutic interventions (if a disease was diagnosed). The researchers observed the confidentiality of data based on the Declaration of Helsinki.

**Data collection:** The participants’ demographic and socioeconomic information, the history of smoking, alcohol and drug use, childhood sexual abuse, STIs, Hepatitis B vaccination, age at the first sexual intercourse, age at starting prostitution, number of sexual intercourses per week, number of clients per week, the use of protection during intercourse, signs and symptoms of STIs in the past year, and experience of sexual violence was collected by one of the researchers via a checklist and in face-to-face interviews. 

**Laboratory assessments**

**ELISA test to diagnose viral infections:** 10 ml of venous blood was taken from each participant and sent to a single laboratory to be tested for HBsAg, HBc Ab, anti-HBs, HCV Ab, and HIV Ab (kit Pishtazteb) 

**Rapid plasma reagin (RPR) test to diagnose syphilis infection:** To perform the test, the BioniK kit was performed according to the manufacturer’s instructions.

**DNA extraction for** ***Papillomavirus*** **:** DNA was extracted, using a kit (High pure PCR Template Preparation; Roche, Germany). The DNA extraction steps were performed according to Kit instructions. Extracted DNA samples were stored in 1.5 ml microtubes at -20°C until PCR. The samples tested in vaginal sex workers were from the cervix and anal sex workers from the anal region.

**PCR assay** **for *Papillomavirus*:** The PCR reactions were performed in a total volume of 25 μL containing PCR Master Mix (CinnaGen, Tehran, Iran). The PCR reactions were performed according to the instructions in the studies of Mohammadpour B et al. and Salehi-Vaziri M et al.’s (14, 15). **Statistical analysis:** The data were analyzed in Stata 14. Qualitative variables were analyzed as frequency (percentage) and quantitative variables as means (standard deviation).

## Results

A total of 138 eligible FSWs had visited the clinic and the affiliated counseling centers from April 2019 to April 2020, of whom 26 women were excluded as they did not consent to participate, and 12 women were excluded because they did not consent to a pap smear and other tests. Finally, 100 women were studied. Table 1 presents an overview of their demographic information and socioeconomic status. The mean age of the participants was 32.8 ± (14.2), ranging from 13 to 50 years. Most of the participants, 40% belonged to the 30 to 40 age group, followed by 35% belonging to the 20 to 30 age group. Most of the FSWs (94%) resided in the city and more than half of the participants (56%) did not have a high-school diploma. Moreover, 23% were illiterate, and only 5% had academic education. 

About 51% reported a history of drug use, of whom, 20% were current drug users at the time of the interview. The most frequently used substance was hashish 62%, followed by methadone 29%. Injecting drug users comprised 14% of the addicted women. The history of alcohol use and smoking was positive in 59% and 57%, respectively. Of those with a positive history of smoking, 38% were current smokers at the time of the interview. A history of imprisonment was reported in 50%. The mean age of the first sexual intercourse was 19.2 ± (6.8) years, 76% had the first sexual intercourse between the ages of 16 to 20 years, and 2% reported the age of the first sexual intercourse to be less than 10 years. Childhood sexual abuse was reported in 28%. The most frequent age range of starting the job was 21 to 25 years (31%), followed by 16 to 20 years (30%). 11% percent had started prostitution when they were 10 to 15 years old. The participants had 4.5 sexual relations per week on average. In terms of protected sex, 31% reported that they always used a condom when having sex with their clients. However, 49% used a condom occasionally, and 20% never used a condom. All new infections were among those who had never used condoms, and two cases of papilloma infection were among those who did not use condoms consistently. As per STIs, 47% of the FSWs reported at least one STI in the past year, with the most prevalent one being vaginal discharge (39%). About half of the women (52%) had never had a pap smear test before the interview, and only 29% had had a pap smear test in the past year. 43% had been vaccinated against hepatitis B, and 6% against HPV. After vaccination against hepatitis B and HPV no one became infected.71% had been tested for HIV in the past 12 months, while 19% did not know about such a test or where to be tested. Two people had coinfection with HCV and HIV and one person HIV, HBV and HCV. Pregnancy occurred in 16% of them because of providing sexual services, of whom 69% had an abortion (table 2).

**Table 1:** Demographic and socioeconomic characteristics of the 100 female sex workers in Kurdistan, Iran, 2019–2020

**Indicator**	**Estimate %**	**95%CI**
Age group		
<20	4%	1.1 – 9.9
20 - 30	35%	25.7 – 45.2
30 - 40	40%	30.3 – 50.3
>40	21%	13.5 – 30.3
Location		
Urban	94%	87.4 – 97.8
rural	6%	2.2 – 12.6
Education		
illiterate	23%	15.2 – 32.5
under diploma	56%	45.7 – 65.9
diploma	16%	9.4 – 24.7
graduated	5%	1.6 – 11.3
Marital status		
single	15%	8.6 – 23.5
married	37%	27.6 – 47.2
Divorced/ widowed	48%	37.9 – 58.2
Having a child		
yes	78%	68.6 – 85.7
no	22%	14.3 – 31.4
Imprisonment history		
yes	50%	39.8 – 60.2
no	50%	39.8 – 60.2
Smoking		
yes	57%	46.7 – 66.9
no	43%	33.1 – 53.3
Alcohol consumption		
yes	59%	48.7 – 68.7
no	41%	31.3 – 51.3

**Table 2:** Risky Behaviors among 100 female sex workers in Kurdistan, Iran, 2019–2020

**Indicator**	**Estimate %**	**95%CI**
Age at first intercourse		
<15	2%	0.2 – 7.0
16 -20	76%	66.4 – 84.0
21 - 25	15%	8.6 – 23.5
26 - 30	6%	2.2 – 12.6
>30	1%	0.0 – 5.4
Age at first commercial sex		
<15	11%	5.6 – 18.8
16 -20	30%	21.2 – 40.0
21 - 25	31%	22.1 – 41.0
26 - 30	13%	7.1 – 21.2
>30	15%	8.6 – 23.5
Number of Clients per week		
1-2	28%	19.5 – 37.9
3-6	38%	28.5 – 48.3
≥6	34%	24.8 – 44.2
Condom use		
Always	31%	22.1 – 41.0
sometimes	49%	38.9 – 59.2
Never	20%	12.7 – 29.2
History of drug use		
Yes	51%	40.8 – 61.1
No	49%	38.9 – 59.2
Drug use at Interview time		
Yes	20%	12.7 – 29.2
No	80%	70.8 – 87.3
injection drug use		
Yes	14%	7.9 – 22.4
No	86%	77.6 – 92.1
History of STI symptoms in the last year		
Yes	47%	36.9 – 57.2
No	53%	42.8 – 63.1
History of HIV testing in the last year		
Yes	71%	61.1 – 79.6
No	29%	20.4 – 38.9
History of pap smear test in the last year		
Yes	29%	20.4 – 38.9
No	71%	61.1 – 79.6
History of HBV vaccination		
Yes	43%	33.1 – 53.3
No	57%	46.7 – 66.9
History of HPV vaccination		
Yes	6%	2.2 – 12.6
No	94%	87.4 – 97.8
Pregnancy record at commercial sex		
Yes	16%	9.4 – 24.7
No	84%	75.3 – 90.6
History of Childhood Sexual Abuse		
Yes	27.8%	19.2 – 37.9
No	72.2%	62.1 – 80.8
Record of physical violence at commercial sex		
Yes	66%	55.8 – 75.2
No	34%	24.8 – 44.2

Of the 100 SWFs, 6 (6%) were infected with HIV (4 were aware of their disease, and 2 were diagnosed in this study); 1 (1%) had hepatitis B (HBsAb), HBC Ab in 4%, and Anti HBsAb in 47% were positive), and 2 (2%) were HCV positive. Only 1 (1%) of women were suspected of syphilis. Based on the PCR test results, HPV was found in 16(16%) samples. Type 16 was observed in 1 sample (6% of HPV positive cases, and 1% of the total studied pap smears), type 18 in 4 samples (25% of the HPV positive cases, and 4% of the total studied pap smears), types 6 and 11 together in 1 sample (6% of the HPV positive cases, and 1% of the total studied pap smears). The other 10 samples had other types of HPV that were not studied (table 3). All HIV-positive women were between 40 and 50 years old. Five of the HIV-positive people had received an HBV vaccine. In one participant, despite being vaccinated, HBsAb had a titer of 7.7, indicating that he was still susceptible to the disease. Another person tested positive for HCV Ab and HBcAb, but tested negative for HBsAg or HBs Ab, indicating HCV infection and a window period or acute exacerbation or past hepatitis B infection. Only one serologically based hepatitis B infection was found to be HBc Ab positive and had a low HBs Ab titer. The 45-year-old woman tested negative for HIV, HCV, and HPV PCR. Four of the women were HBc Ab positive (one had a low HBs Ab but a positive HBs Ab and HBs Ag. The second was HIV and HCV positive, already infected with HBV but HBs Ab not yet elevated. The third person had only HBc Ab and HBs Ab positive, which may have been first infected and then converted to HBsAg. The fourth person had HBs Ab, HBs Ag, and HBc Ab positive this finding can indicate that the patient has been infected with hepatitis B virus, but it was the beginning of the conflict and will gradually disappear with the increase of the antigen antibody. That is, if the patient's test is repeated after a few weeks, HBsAg will be negative and HBsAb and HBcAb will be positive.

**Table 3:** Estimate % and 95% CI for HIV, HBV, HCV, HPV and Syphilis of female sex workers, Kurdistan, Iran, 2019–2020

**Indicator**	**Estimate %**	**95%CI**
HIV Positive	6%	2.2 – 12.6
HBV Positive	1%	0.0 – 5.4
HCV Positive	2%	0.2 – 7.0
HPV Positive	16%	9.4 –24.7
RPR Positive	1%	0.0 – 5.4

## Discussion

This study examined the prevalence of viral infections in the FSW population and provides information on lifestyle and high-risk behaviors. As prostitution is illegal in Iran, FSWs do not receive services and scant information is available on them; the results of this study can, therefore, help enact policies to prevent and control the transmission of STIs in society. Based on the findings, 6% of the FSWs had HIV, 1% had hepatitis B, 2% had hepatitis C, and 16% had HPV. All of the infected FSW with HIV, HBV, and HCV had history of drug abuse.

Other studies conducted in the past decade have reported the HIV prevalence to be 0–5% among FSWs in Iran (16-18). In a 2013 study in Shiraz (in a southern Iranian province), the prevalence of HIV among FSWs was 4.7%. 70% reported drug use, of whom only 16% were injection drug users (IDUs). Moreover, unprotected sex was reported to be 24% in the previous months (18). In another study conducted in Tehran in 2012–2013, 5% of the FSWs were infected with HIV. In this study, 65.2% of the FSWs had used a condom in their last sexual intercourse, while only 49% used a condom in all their sexual relations. Moreover, 33% had been tested for HIV in the last 12 months and knew the results. Furthermore, 91% had taken narcotics at least once, 56% were active substance users at the time of the interview, and 25% were injecting drug users (IDUs) (19). In Mirzazadeh et al.’s study, the prevalence of HIV was 5%, HPV 41.8%, gonorrhea 1.3%, trichomonas 11.9%, chlamydia 6%, and syphilis 0.4–4% (20). In another study, Haraz Wong et al. indicated that the prevalence of syphilis was 2.1% and none of the people were HIV positive. The prevalence of positive test for pharyngeal chlamydia and Neisseria gonorrhoeae was 3.2% and 4.4%, respectively. However, the prevalence of urinary tract chlamydia infection and gonococcal infection were 10.6% and 0.9%, respectively (21).

 In Michel Decker et al.’s study indicated that in Russian for 147 FSWs, the prevalence of HIV was 4.8%, the prevalence of chlamydia was 15%, syphilis was 11.6%, and gonorrhea was 6.8%. People ranged in age from 17 to 40. More than half (53.7%) were over 22 years old. The prevalence of sexually transmitted infections and HIV together was higher in immigrant prostitutes than in indigenous women at 42.6% and 17.4%, respectively (22).

The findings of the present study indicate that the prevalence of HIV among FSWs in this region is higher than that reported by other studies in Iran. Compared to previous studies, a higher percentage of FSWs in the current study had been tested for HIV in the last 12 months and knew the results. Moreover, fewer FSWs in this study reported using narcotics at least once, were active users, or were IDUs. Nevertheless, the indices related to unprotected sex found in the current study were higher than other studies. In HIV/AIDS modeling, Haghdoost et al. (2014) reported that the transmission pattern has shifted from unsafe injection towards unprotected sex (20). In our study, unprotected sex was the most high-risk behavior in terms of STIs transmission in this group. Based on these results, the FSWs in these regions have little awareness of HIV prevention methods. Healthcare information disseminating organizations must, therefore, raise this high-risk group’s awareness, and provide free and easy access to condoms and other forms of protection due to FSWs’ low socioeconomic status. In our results, 43 cases were vaccinated, 27 of which did not have an adequate antibody titer, which was due to the vaccine injection in the last 5 to 10 years and suggest that we should check the vaccine titer annually. 

In our study, the prevalence of hepatitis B (1%) did not differ from that in the general population of Iran (1.09%) (23), but the prevalence of hepatitis C (2%) was higher than that in the general population of this country (0.16%) (24). In a study conducted in Isfahan on 100 women who had illegal sexual relations, the prevalence of hepatitis B and C was 1 and 10%, respectively (25). In a study in Tehran, the prevalence of hepatitis B and C was respectively 1 and 8% (26). These results are consistent with our study in terms of hepatitis B prevalence, but hepatitis C prevalence was much lower in our study than the cited ones. The high vaccination rate of the FSWs in the current study (43%) may explain the low prevalence of hepatitis B in them, showing that vaccination is the best strategy for mitigating the risk of HBV infection. The findings of the present study highlight the necessity of preventive interventions for this high-risk group to prevent HCV transmission in society. 

In the present study, the HPV prevalence among FSWs was 16%, and the frequency of the 16 and 18 genotypes with a high oncogenic risk was 1% and 4%, respectively. A systematic review and meta-analysis on 14 studies in Iran reported the prevalence of HPV among healthy women in different Iranian provinces to be 9.4%, and the prevalence of HPV types 16 and 18 as 2.3% and 1.7%, respectively (27). Based on the latest reports published by the ICO HPV INFORMATION CENTRE in 2019 about the HPV status in Iran, the prevalence of HPV in the studied women with normal cytology was 7.4%. HPV type 16 existed in 2.2%, and type 18 in 0.6% of women with normal cervical cytology. Overall, about 2.8% of women in the general population carried HPV 16/18 in their cervical discharge (28). Therefore, the frequency of genotype 18 in the FSWs studied in the current study was higher than the general population of other cities. The high prevalence of oncogenic HPV in this study is similar to the findings of a study on a group of FSWs in Turkey; which reported HPV prevalence to be 20.1% and the HPV type 18 was the most prevalent genotype identified among the FSWs (40% of HPV positive women) (29). Due to the high prevalence of high-risk types in these patients, which is strongly correlated with cervical cancer, preventive measures, vaccination, and regular cervical cancer screening programs must be implemented for this vulnerable group. 

The main limitation of this study was that most of the studied women had a low socioeconomic status. The findings should, therefore, be generalized to all FSWs in society with caution. Moreover, we did not determine the other HPV genotypes; the laboratory in which the samples were tested only had access to the positive control for genotypes 6, 11, 16, and 18, and the other genotypes could not be detected. 

This study was conducted for the first time in west of Iran, showed a high prevalence of viral infection among FSWs. High-risk and unprotected sexual behaviors, which are strongly correlated with the prevalence of these infections, are also considerably prevalent in this group. These findings indicate that FSWs are a high-priority group for regular screening programs, and national HIV and cervical cancer prevention programs should preferably be concentrated on these women. Raising their awareness and educating them on how to prevent STIs and offering efficient health insurance that provides easy access to healthcare can effectively reduce the prevalence of these infections among FSWs.
